# GLI1 activation by non-classical pathway integrin α_v_β_3_/ERK1/2 maintains stem cell-like phenotype of multicellular aggregates in gastric cancer peritoneal metastasis

**DOI:** 10.1038/s41419-019-1776-x

**Published:** 2019-07-31

**Authors:** Hui Dong, Hongchang Liu, Wen Zhou, Fan Zhang, Chuan Li, Jun Chen, Chenjun Tan, Bo Tang, Peiwu Yu

**Affiliations:** 1 Department of General Surgery, Center of Minimal Invasive Gastrointestinal Surgery, Southwest Hospital, Army Medical University (Third Military Medical University), 400038 Chongqing, China; 2 Department of Biological Sciences, Columbia University, New York, NY 10027 USA

**Keywords:** Gastric cancer, Gastric cancer

## Abstract

Peritoneal metastasis is one of the most important causes of postoperative death in patients with gastric cancer, and the exact mechanism remains unclear. The proliferation of multicellular aggregates of exfoliated malignant gastric cells in the abdominal cavity is the focus of current research. However, the mechanism how gastric cancer multicellular aggregates survive remains unclear. In this study, we demonstrated that multicellular aggregates of exfoliated gastric cancer cells in the abdominal cavity expressed a stem cell-Like phenotype. We found that Integrin α_v_β_3_ not only mediated adhesion of gastric cancer multicellular aggregates to form independent functional units, but also maintained their stem cell-like phenotype by the non-classical pathway Integrin α_v_β_3_/ERK1/2/GLI1. In addition, ERK1/2 directly regulates the transcriptional activity of GLI1. GLI1 is a key effector of the Integrin α_v_β_3_ pathway in regulating stem cell-like phenotype in multicellular aggregates. Our data indicates that although there is a crosstalk between the non-classical Integrin α_v_β_3_ pathway and the classical Hedgehog pathway, the activation of GLI1 is almost independent of the Hedgehog pathway in multicellular aggregates of gastric cancer cells. Our study provides a basis for blocking GLI1 activity in the prevention and treatment of peritoneal metastases of gastric cancer.

## Introduction

Gastric cancer (GC) is the third leading cause of cancer-related deaths worldwide^1,2^. Peritoneal metastasis is the most common metastasis observed in GC patients after surgery^3–5^. According to the classic “seed and soil” theory^6,7^, the survival of exfoliated cancer cells from the primary site to the abdominal cavity is an important step of GC peritoneal metastasis^8^. Two common types of exfoliated cancer cells are scattered-free cancer cells and multiple exfoliated cancer cells. The latter forms multicellular aggregates/spheroids (MCAs/MCSs). Scattered-free cancer cells often undergo anoikis when nutrition is relatively scarce, while MCAs can be suspended and grown in the abdominal microenvironment^9,10^. MCAs of GC cells are the major seeding cells of peritoneal metastases, but the survival mechanism of MCAs remains unclear. In our study, we found that MCAs of GC cells possessed several cancer stem cell-like phenotypes, including colony formation, cancer stem cell marker gene expression and tumorigenesis in vivo. Therefore, we investigated the mechanism of maintaining cancer stem cell-like phenotype.

The classical Hedgehog-GLI signaling pathway plays an important role in the regulation of the stemness of cancer cells^11–13^. GLI1 is not only a downstream key effector of the classical Hedgehog (Hh) ligands-PTCH1-SMO axis, but also has crosstalk with the non-classical PI3K/AKT, TNF-α/mTOR, and MAPK/ERK1/2 pathways^14^. Our results showed that the mRNA expression of stemness-related markers in peritoneal MCAs of exfoliated GC cells was slightly decreased after treatment with inhibitors of the classical Hedgehog pathway, whereas the addition of GLI1 or ERK1/2 inhibitors resulted in a significant decrease. These results suggest that the stem cell-like phenotype of gastric cancer MCAs may be regulated by the activation of GLI1 via the ERK1/2 pathway. The specific regulatory pathway needs to be studied further.

Integrin is a heterodimer formed by α and β subunits. Integrins are distributed and function differently in different tissues. The β_3_ subunit mediates tumor cell aggregation and cell viability^15^. Our results indicated that Integrin α_v_β_3_ mediates the aggregation of exfoliated GC cells to form MCAs in the abdominal cavity. GLI1 often functions as a downstream regulatory molecule of the Integrin signaling pathway^16^.

Here, we present evidence that the non-classical pathway Integrin α_v_β_3_/ERK1/2/GLI1 maintains the stem cell-like characteristic of MCAs in GC peritoneal metastasis. GLI1, as a downstream key effector of the non-classical Integrin α_v_β_3_ pathway, plays an important role in the regulation of stem cell-like phenotypes. These results may explain why gastric cancer MCAs can maintain a stemness phenotype in the abdominal cavity.

## Results

### Peritoneal MCAs of exfoliated GC cells expressing a stem cell-like phenotype

**Fig. 1:**
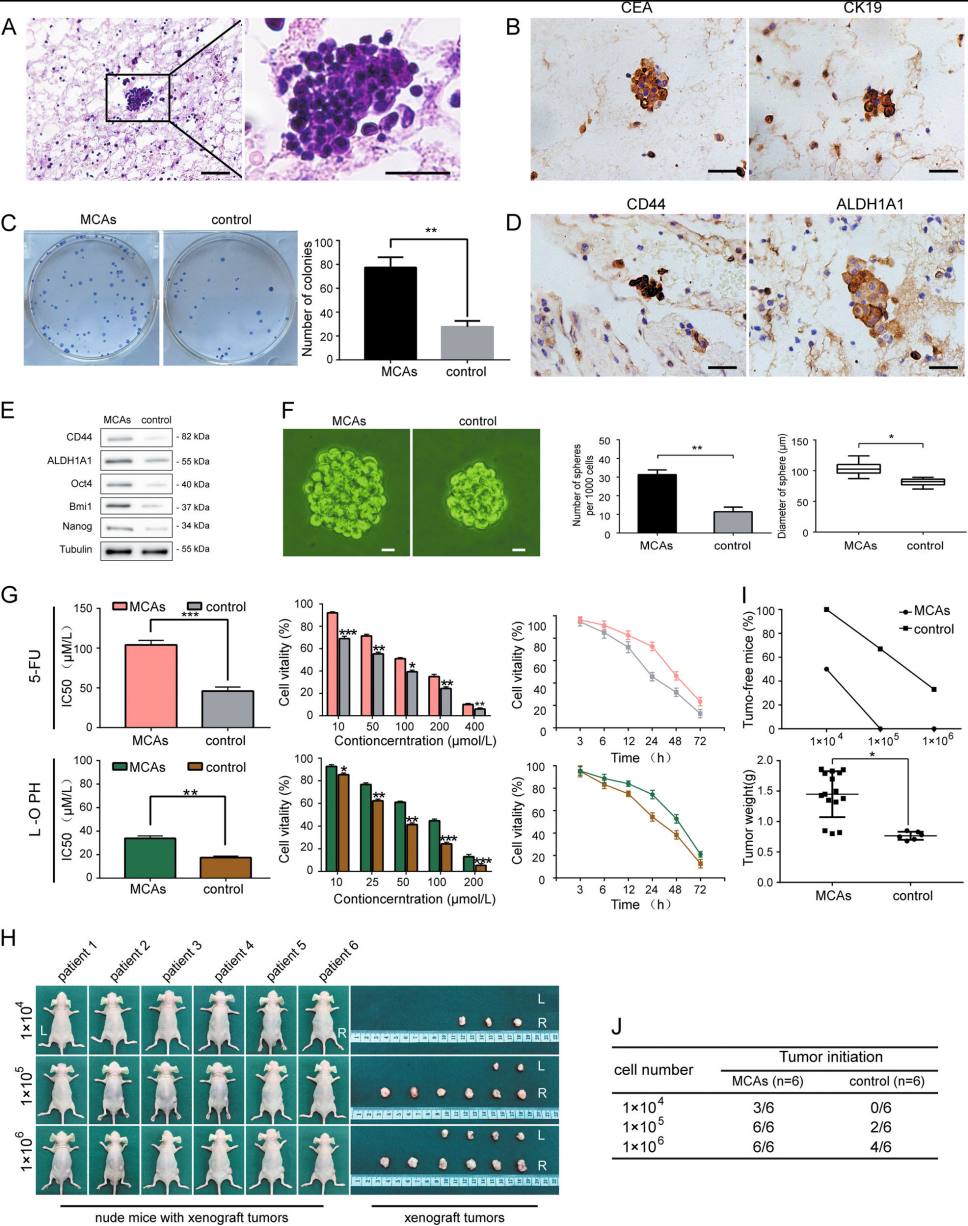
Peritoneal MCAs of exfoliated GC cells expressing a stem cell-like phenotype. **a** Representative H&E images of peritoneal MCAs and scattered-free cancer cells. Scale bar = 100 μm. **b** Representative IHC images of CEA and CK19 in peritoneal MCAs and scattered-free cancer cells. Scale bar = 100 μm. **c** Higher colony forming ability of peritoneal MCAs than scattered-free cancer cells in the control group. **d**, **e** IHC and western blottingting showing upregulated stemness-related genes CD44, ALDH1A1, Oct4, Bmi1 and Nanog expressed by peritoneal MCAs compared to scattered-free cancer cells in the control group. Scale bar = 100 μm. **f** Increased tumor spheres forming ability of peritoneal MCAs compared to scattered-free cancer cells in the control group. Scale bar = 10 μm. **g** Higher cell viability of peritoneal MCAs after being treated with different concentrations and for different durations with 5-fluorouracil and oxaliplatin compared to scattered-free cancer cells in the control group. **h** Formation of subcutaneous xenograft tumors by peritoneal MCAs (R) than scattered-free cancer cells (L). **i**, **j** Increased number and weight of xenograft tumors formed by peritoneal MCAs compared to scattered-free cancer cells in the control group. Each bar in the figure represents the mean ± SEM of triplicates. *p < 0.05; **p < 0.01; ***p < 0.001

To identify the biological function of peritoneal MCAs of exfoliated GC cells, we collected samples of ascites/peritoneal lavage fluid from 14 patients who suffered from peritoneal metastasis after gastric cancer surgery. These patients did not undergo chemotherapy or radiotherapy before surgery. The H&E staining (Fig. 1a) and immunohistochemistry (Fig. 1b) showed that peritoneal MCAs and scattered-free cancer cells both expressed carcinoembryonic antigen (CEA) and adenocarcinoma marker CK19, which are the markers of epithelium originated.

Cancer stem cell-like biological characteristics include self-renewal, proliferation, drug resistance, tumorigenesis, and stemness-related markers^17,18^. Cell colony formation assays (Fig. 1c) showed that peritoneal MCAs of exfoliated GC cells had increased colony forming efficiency compared with scattered-free cancer cells in the control group; The assays indicated that peritoneal MCAs of exfoliated GC cells have a stronger capacity for self-renewal. We further examined the levels of stemness-related markers. The immunohistochemistry results and Flow cytometry-based cell sorting (Fig. 1d, Supplementary Fig. S4e) showed positive expression of CD44^19,20^ and ALDH1A1 in peritoneal MCAs of exfoliated GC cells; The Western blotting results (Fig. 1e) also showed high levels of stemness-related genes, including CD44, ALDH1A1, Oct4, Bmi1 and Nanog^21^, in the peritoneal MCAs of exfoliated GC cells compared with scattered-free cancer cells. In addition, similar results were obtained in a serum-free suspension culture (Fig. 1f). The number of tumor spheres formed by peritoneal MCAs of exfoliated GC cells was larger than that of scattered-free cancer cells; And the diameter of tumor spheres formed by scattered-free cancer cells was smaller than peritoneal MCAs, which suggests peritoneal MCAs have stronger proliferative capacity. To determine the sensitivity of peritoneal MCAs of exfoliated GC cells to the first-line chemotherapy agents 5-fluorouracil and oxaliplatin, we performed CCK8 assays (Fig. 1g). The results showed that peritoneal MCAs had stronger drug resistance over time. The percentage of peritoneal MCAs increased proportionally to the increased concentration of chemotherapeutic agents within a 72-h period. Efficient tumorigenesis in vivo is the most important feature of cancer stem cells^22^. We selected 6 samples of peritoneal MCAs from 14 samples to perform a tumorigenicity assays in nude mice (Fig. 1h–j). The assays showed that the number and weight of the xenograft tumors formed by peritoneal MCAs of exfoliated GC cells were always higher than those formed by scattered-free cancer cells. This result suggests that peritoneal MCAs have extremely high tumorigenicity. Together, these data suggest that peritoneal MCAs of exfoliated GC cells may express stem cell-like characteristics.

### GLI1 activation via the non-classical pathway Integrin α_v_β_3_/ERK1/2 in MCAs of GC cells

**Fig. 2:**
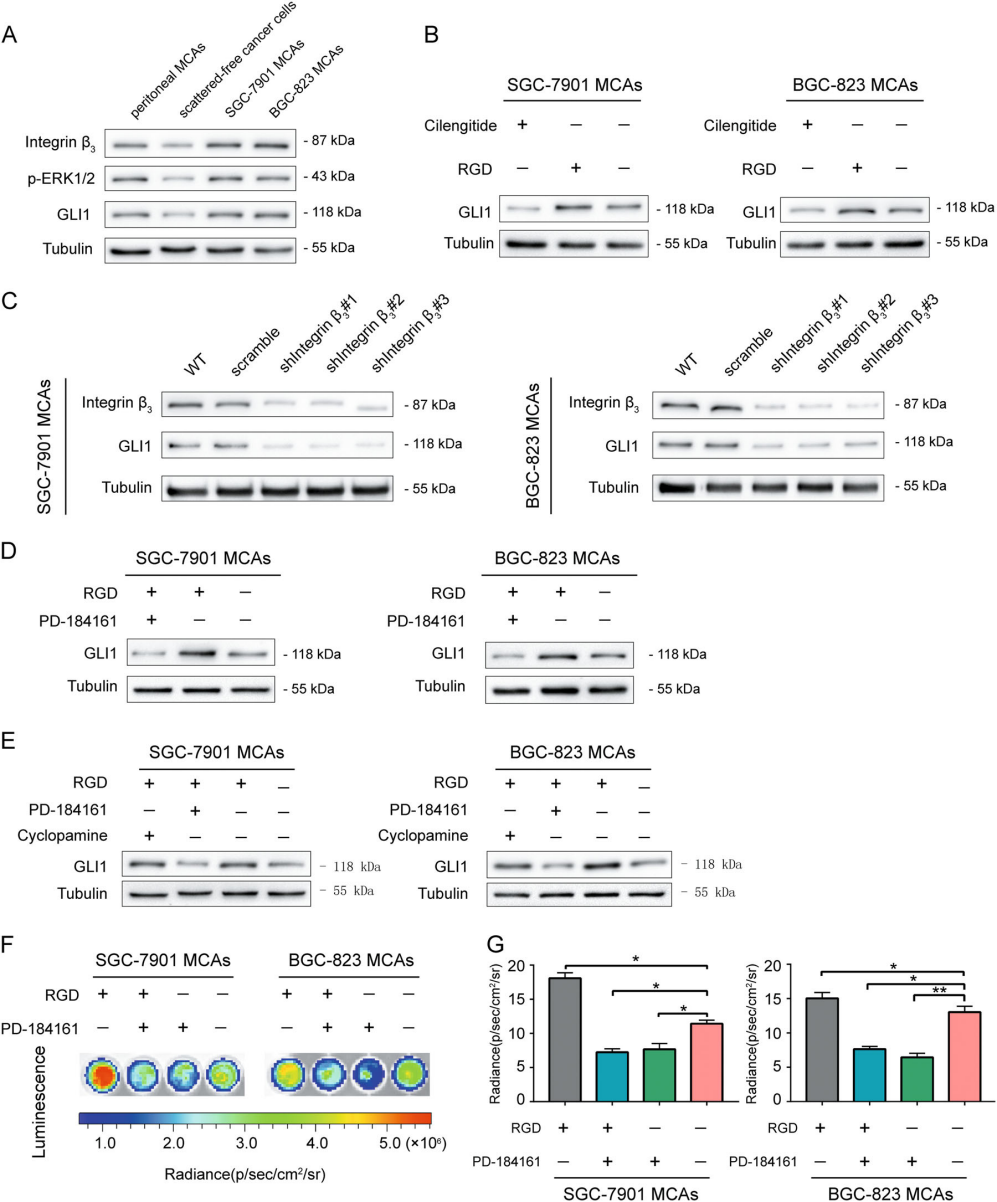
GLI1 activation via the non-classical pathway Integrin α_v_β_3_/ERK1/2 in MCAs of GC cells. **a** Western blottingting showing upregulated Integrin α_v_β_3_, p-ERK1/2 and GLI1 in peritoneal MCAs, BGC823 MCAs and SGC7901 MCAs compared to scattered-free cancer cells in the control group. **b** Western blottingting showing downregulated GLI1 with Integrin α_v_β_3_ inhibitor Cilengitide and upregulated GLI1 with co-stimulator ligand RGD compared to the blank control in SGC7901 MCAs and BGC823 MCAs. **c** Western blottingting showing the most pronounced effect of Integrin β_3_ silencing in the shIntegrin β_3_#1 group. **d** Western blottingting showing downregulated GLI1 with Integrin α_v_β_3_ co-stimulator ligand RGD plus ERK1/2 inhibitor PD-184161 and upregulated GLI1 with RGD alone compared to the blank control in SGC7901 MCAs and BGC823 MCAs. **e** Western blottingting showing slightly decreased GLI1 with Hedgehog/Smoothened pathway inhibitor Cyclopamine compared with the blank control group in SGC7901 MCAs and BGC823 MCAs. **f**, **g** Decreased luminescence of GLI1 in the group of Integrin α_v_β_3_ co-simulator ligand RGD plus ERK1/2 inhibitor PD-184161 or PD-184161 alone compared to the blank control. Each bar in the figure represents the mean ± SEM of triplicates. *p < 0.05; **p < 0.01

In order to further study the underlying molecular mechanism of peritoneal MCAs stemness, we first established serum-free suspension culture derivatives from two gastric cell lines, SGC7901 and BGC823. These suspension culture derivatives resembled the morphology of peritoneal MCAs^23–26^, therefore we named them SGC7901 MCAs and BGC823 MCAs. Similar to the results we obtained in peritoneal MCAs from patient samples, the protein levels of Integrin α_v_β_3_, p-ERK1/2, and GLI1 were higher in BGC823 MCAs and SGC7901 MCAs than in scattered-free cancer cells (Fig. 2a).

Our results indicates that Integrin α_v_β_3_ mediates cell aggregates and maintains the viability of peritoneal MCAs in GC cells (Supplementary Fig. S1a–c).To determine whether Integrin α_v_β_3_ regulates GLI1, Integrin α_v_β_3_ inhibitor Cilengitide, co-stimulator ligand RGD and a blank control DMSO were added to BGC823 MCAs and SGC7901 MCAs, respectively. The Western blotting result (Fig. 2b) showed that the change in the activity of Integrin α_v_β_3_ positively correlates with the protein levels of GLI1. In complimentary to small molecule inhibitor study, we used lentivirus to silence Integrin β_3_ (Fig. 2c). Then we found that the protein level of GLI1 was significantly decreased, especially in the shIntegrin β_3_#1 group. Results showed decreased GLI1 phosphorylation in the shIntegrin β_3_#1 group compared to the blank control in BGC-823 MCA cells (Supplementary Fig. S6b).These results indicated that Integrin α_v_β_3_ can specifically regulate GLI1 in gastric cancer MCAs.

Next, to determine whether Integrin α_v_β_3_ regulates GLI1 activity through ERK1/2 pathway, BGC823 MCAs and SGC7901 MCAs were treated with either an Integrin α_v_β_3_ co-stimulator ligand RGD alone, or in combination with ERK inhibitor PD-184161. Western blotting was used to detect GLI1 protein levels (Fig. 2d). In the group treated with Integrin α_v_β_3_ co-stimulator ligand and an ERK inhibitor, the protein levels of GLI1 was still decreased even though Integrin α_v_β_3_ was stimulated. This suggests that Integrin α_v_β_3_ regulates GLI1 through ERK1/2 pathway.

The classical pathway of GLI1 activation is the Hh ligands-PTCH1-SMO axis^27,28^. To test whether the classical Hedgehog-GLI signaling pathway, in addition to the non-classical pathway Integrin α_v_β_3_/ERK1/2, regulates GLI1 in gastric cancer MCAs, we pretreated BGC823 MCAs and SGC7901 MCAs with Integrin α_v_β_3_ co-stimulator ligand RGD. Then we added either the Hedgehog/Smoothened pathway inhibitor Cyclopamine, ERK inhibitor PD-184161 or a blank control DMSO in each group, respectively. GLI1 expression was detected by Western blottingting (Fig. 2e). The protein level of GLI1 was slightly decreased after inhibition of the Hedgehog/Smoothened pathway compared with the blank control group, whereas it was significantly decreased in ERK inhibition group. In complimentary to small molecule inhibitor study, we used lentivirus to silence Smo (Supplementary Fig. S5a).Consistent results were detected in SGC7901 MCAs and BGC823 MCAs (Supplementary Fig. S5b, c).These results indicate that although crosstalk with the classic Hedgehog-GLI signaling pathway exists, GLI1 activation occurs mainly through the non-classical pathway Integrin α_v_β_3_/ERK1/2 in MCAs of GC cells.

To further investigate whether ERK1/2 directly regulated the transcriptional activity of GLI1, we treated BGC823 MCAs and SGC7901 MCAs transduced with the luciferase reporter gene vector containing the GLI1 promoter region with either Integrin α_v_β_3_ co-stimulator ligand RGD only, Integrin α_v_β_3_ co-stimulator ligand RGD plus ERK1/2 inhibitor PD-184161 or ERK1/2 inhibitor PD-184161 only. Three days later we measured the luminescence in each group (Fig. 2f, g). Compared with the blank control group, the luminescence of GLI1 treated with Integrin α_v_β_3_ co-simulator ligand plus ERK1/2 inhibitor was significantly decreased, and similar results were seen in the group of ERK1/2 inhibitor alone. Western blottingting also showed decreased GLI1 targets c-Myc and Cyclin D1 in the group of Integrin α_v_β_3_ co-simulator ligand RGD plus ERK1/2 inhibitor PD-184161 or PD-184161 alone compared to the blank control (Supplementary Fig. S5d).In addition, we found decreased GLI1 phosphorylation in SGC-7901 or BGC-823 MCA cells treated with the selective ERK1/2 inhibitor in BGC823 MCAs (Supplementary Fig. S6a). This suggests that GLI1 in MCAs of GC cells may be directly regulated by ERK1/2.

In the same experimental groups as above, the gene levels of GLI1 detected by Real-time PCR in peritoneal MCAs, SGC7901 MCAs and BGC823 MCAs were similar (Supplementary Fig. S3a–i), and were consistent with the above protein levels of SGC7901 MCAs and BGC823 MCAs. These results indicate that GLI1 is mainly regulated through the non-classical pathway Integrin α_v_β_3_/ERK1/2 in MCAs of gastric cancer cells.

### The Integrin α_v_β_3_/ERK1/2/GLI1 pathway maintains the stem cell-like phenotype in MCAs of GC cells

**Fig. 3:**
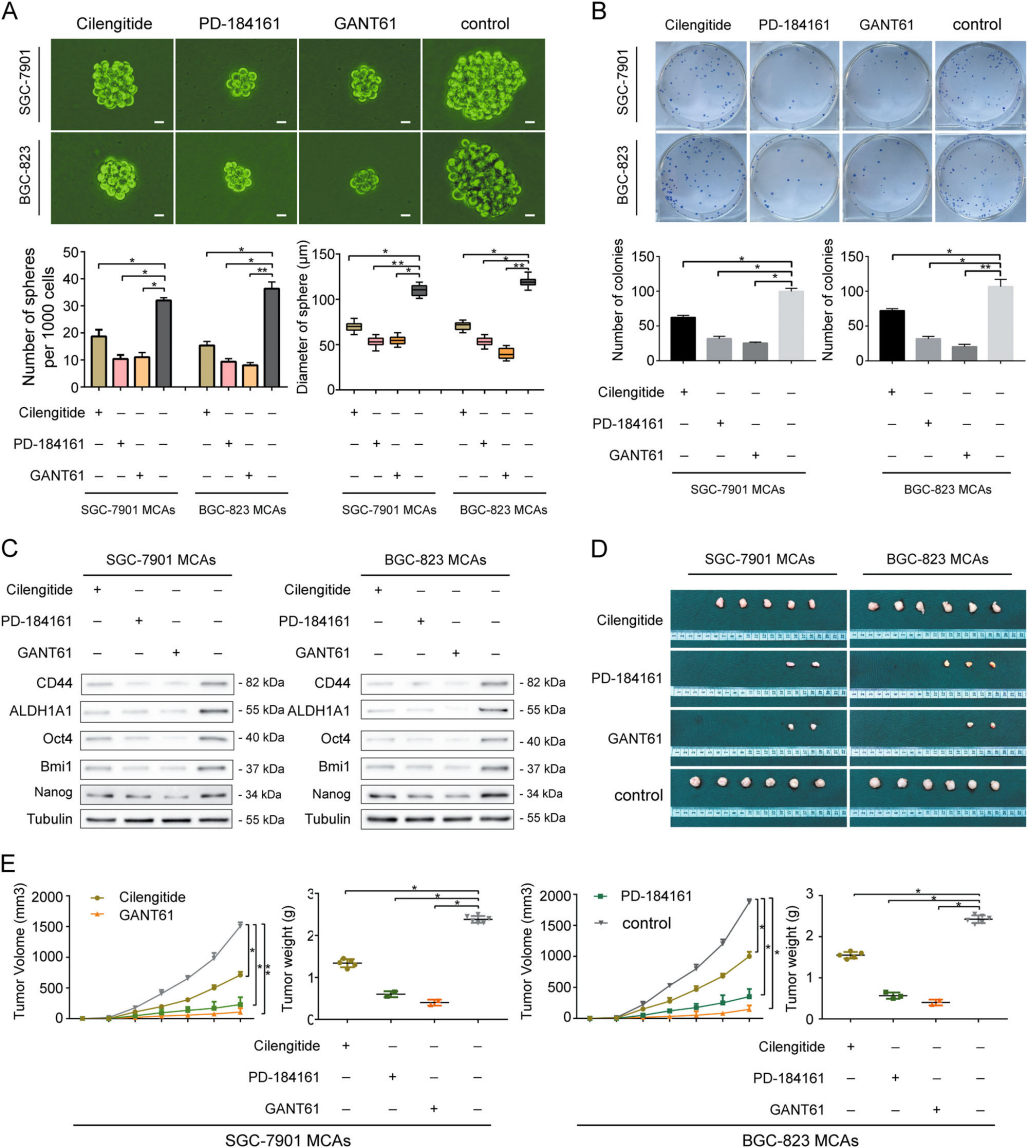
The Integrin α_v_β_3_/ERK1/2/GLI1 pathway maintains the stem cell-like phenotype in MCAs of GC cells. **a** Decreased tumor spheres forming ability in the group of Integrin α_v_β_3_ inhibitor Cilengitide or ERK1/2 inhibitor PD-184161 or GLI1 inhibitor GANT61 compared to the blank control in SGC7901 MCAs and BGC823 MCAs. Scale bar = 10μm. **b** Lower colony forming ability in the group of Integrin α_v_β_3_ inhibitor Cilengitide or ERK1/2 inhibitor PD-184161 or GLI1 inhibitor GANT61 compared to the blank control in SGC7901 MCAs and BGC823 MCAs. **c** Western blottingting showing downregulated stemness-related genes CD44, ALDH1A1, Oct4, Bmi1 and Nanog in the group of Integrin α_v_β_3_ inhibitor Cilengitide or ERK1/2 inhibitor PD-184161 or GLI1 inhibitor GANT61 compared to the blank control in SGC7901 MCAs and BGC823 MCAs. **d**, **e** Decreased volume and weight of xenograft tumors in the group of Integrinα_v_β_3_ inhibitor Cilengitide or ERK1/2 inhibitor PD-184161 or GLI1 inhibitor GANT61 compared to the blank control in SGC7901 MCAs and BGC823 MCAs. Each bar in the figure represents the mean ± SEM of triplicates. *p < 0.05; **p < 0.01

To investigate whether the Integrin α_v_β_3_/ERK1/2/GLI1 pathway regulates the stem cell-like characteristics of gastric cancer MCAs, Integrin α_v_β_3_ inhibitor Cilengitide, ERK inhibitor PD-184161, GLI1 inhibitor GANT61 and a blank control DMSO were added into BGC823 MCAs and SGC7901 MCAs. The stem cell-like characteristics of four experimental groups were examined for proliferation, self-renewal, the protein levels of stemness-related markers, and tumorigenesis. In contrast to the blank control group, the stemness characteristics of GC MCAs in other three groups were significantly reduced after the inhibition of Integrin α_v_β_3_, ERK1/2, and GLI1, respectively. Grown in serum-free suspension culture (Fig. 3a), there were fewer tumor spheres, which are also with smaller diameters, which suggests that the proliferation ability of these three groups with inhibitors decreased. The cell colony formation assay (Fig. 3b) showed that the colony numbers in each group after inhibiting Integrin α_v_β_3_, ERK, and GLI1 were significantly reduced, indicating that their self-renewal ability decreased. Further experiments demonstrated corresponding changes in Western blotting analysis (Fig. 3c). The result showed that the protein levels of the stemness-related markers Bmi1, Oct4, Nanog, CD44 and ALDH1A1 were significantly downregulated in these three groups with inhibitors. To verify this control scheme, we also tested the gene expression of GLI1 in each group. The Real-time PCR result (Supplementary Fig. S1d) showed decreased GLI1 in in these three groups with inhibitors. In addition, the tumorigenicity assay in nude mice (Fig. 3d) showed that the number and the volume of xenograft tumors (Fig. 3e) decreased in each group after inhibiting Integrin α_v_β_3_, ERK, and GLI1, indicating a significant reduction in tumorigenesis. Consistent results were detected for proliferation, self-renewal and the gene levels of stemness-related markers in peritoneal MCAs compared to SGC7901 MCAs and BGC823 MCAs (Supplementary Fig. S3j-l). These results suggest that the inhibition of any molecule in the Integrin α_v_β_3_/ERK1/2/GLI1 pathway can downregulate the stem cell-like characteristics in MCAs of gastric cancer cells. And the reduction in the stem cell-like phenotype was the most profound in the GLI1 inhibitor group.

### The key effector GLI1 of Integrin α_v_β_3_/ERK1/2 pathway regulates the stem cell-like phenotype in MCAs of GC cells

**Fig. 4:**
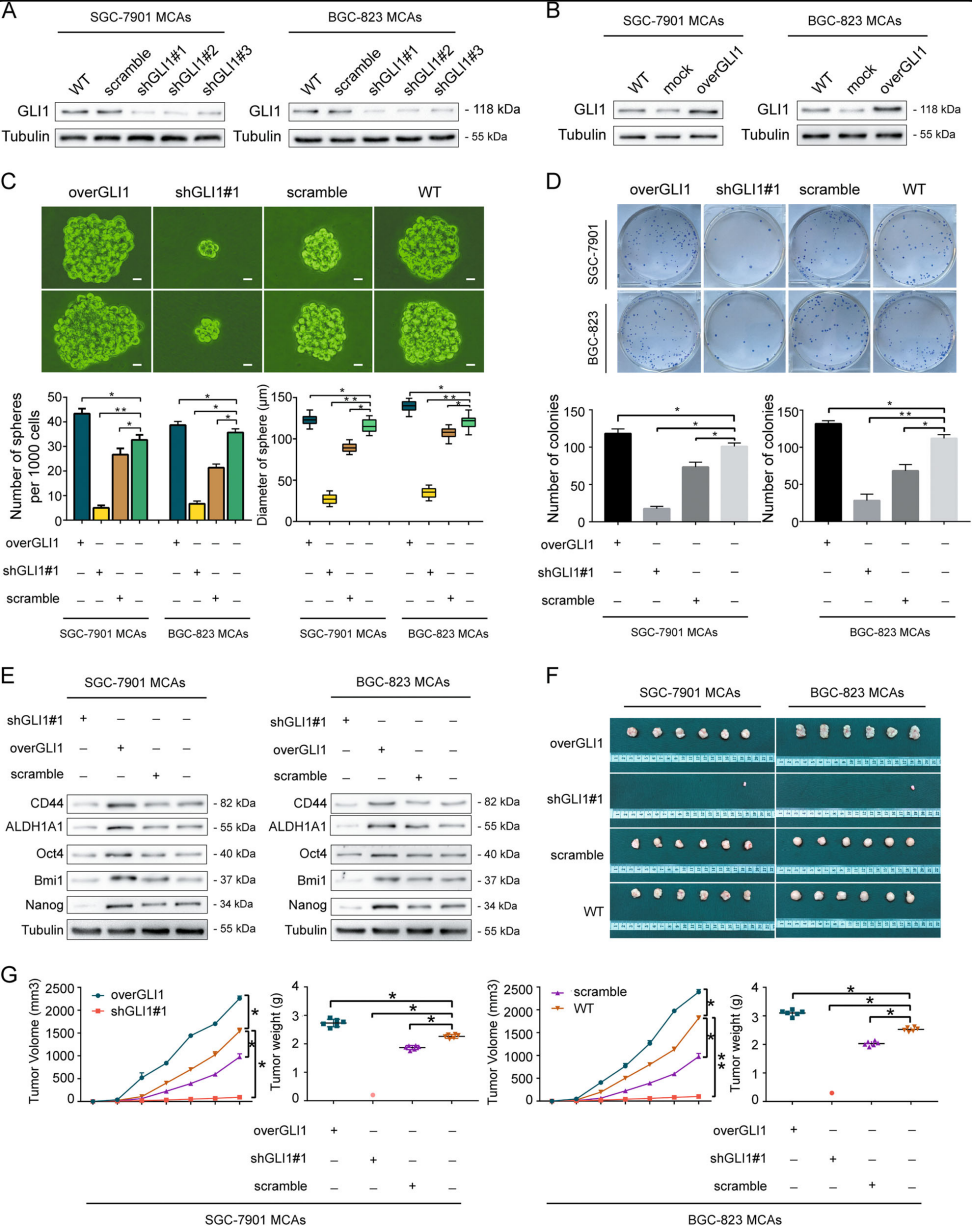
The key effector GLI1 of Integrin α_v_β_3_/ERK1/2 pathway regulates the stem cell-like phenotype in MCAs of GC cells. **a** Western blottingting showing the most pronounced effect of GLI1 silencing in the shGLI1#1 group in BGC823MCAs and SGC7901MCAs. **b** Western blottingting showing evident overexpression of GLI1 in BGC823MCAs and SGC7901MCAs transduced with overGLI1 lentivirus. **c** Increased tumor spheres forming ability in the overGLI1 group and decreased formating ability in the shGLI1#1 group compared to the control group (WT) in SGC7901 MCAs and BGC823 MCAs. Scale bar = 10 μm. **d** Higher colony forming ability in the overGLI1 group and lower colony forming ability in the shGLI1#1 group compared to the control group in SGC7901 MCAs and BGC823 MCAs. **e** Western blottingting showing downregulated stemness-related genes in the shGLI1#1 group but upregulated genes CD44, ALDH1A1, Oct4, Bmi1 and Nanog in the overGLI1 group compared to the control group in SGC7901 MCAs and BGC823 MCAs. **f**, **g** Decreased volume and weight of xenograft tumors in the shGLI1#1 group and increased volume and weight in the overGLI1 group compared to the control group in SGC7901 MCAs and BGC823 MCAs. Each bar in the figure represents the mean ± SEM of triplicates. *p < 0.05; **p < 0.01

Because GLI1 is a key downstream effector of the crosstalk between the unclassical Integrin α_v_β_3_ pathway and the classical Hedgehog pathway, it is critical to study the role of GLI1 on the regulation of the stem cell-like biological characteristics in MACs of GC cells. BGC823 MCAs and SGC7901 MCAs were either transduced with either GLI1 silencing virus (Fig. 4a), a negative shRNA control shRNA virus, or transduced with GLI1 overexpressing vectors (Fig. 4b), and an empty pcDNA3 vector, respectively. Then the stem cell-like characteristics of each experimental group was examined for proliferation, self-renewal, the protein levels of stemness-related markers, and tumorigenesis. The stem cell-like biological characteristics in the shGLI1#1 group were significantly reduced compared with the control group. A smaller number of tumor spheres with smaller diameters were formed in the shGLI1#1 group in serum-free suspension culture (Fig. 4c), suggesting a decrease in the ability of proliferation. The colony formation assay data (Fig. 4d) showed a decrease in the number of colonies in the shGLI1#1 group compared with the control group, reflecting a decrease in the ability of self-renewal; Western blotting analysis (Fig. 4e) showed that the protein levels of stemness-related markers Bmi1, Oct4, Nanog, CD44, and ALDH1A1 were significantly decreased in the shGLI1#1 group. To verify this control scheme, we also tested the gene expression of GLI1 in each group. The Real-time PCR result (Supplementary Fig. S2e) showed decreased GLI1 in the shGLI1#1 group compared with the control group. The tumorigenicity assay in nude mice (Fig. 4f, g) showed that both the number and volume of xenograft tumors formed in the shGLI1#1 group were reduced, indicating a decrease in the ability of tumorigenesis. In contrast, the stem cell-like biological characteristics in the overGLI1 group significantly increased. These findings suggest that GLI1 is the key molecule that regulates the stem cell-like biological characteristics of gastric cancer MCAs through the Integrin α_v_β_3_/ERK1/2/GLI1 pathway. Once GLI1 was knocked down, the stem cell-like phenotype of gastric cancer MCAs decreased accordingly.

### In vivo validation of the role of the Integrin α_v_β_3_/ERK1/2/GLI1 pathway on the peritoneal metastasis of GC

**Fig. 5:**
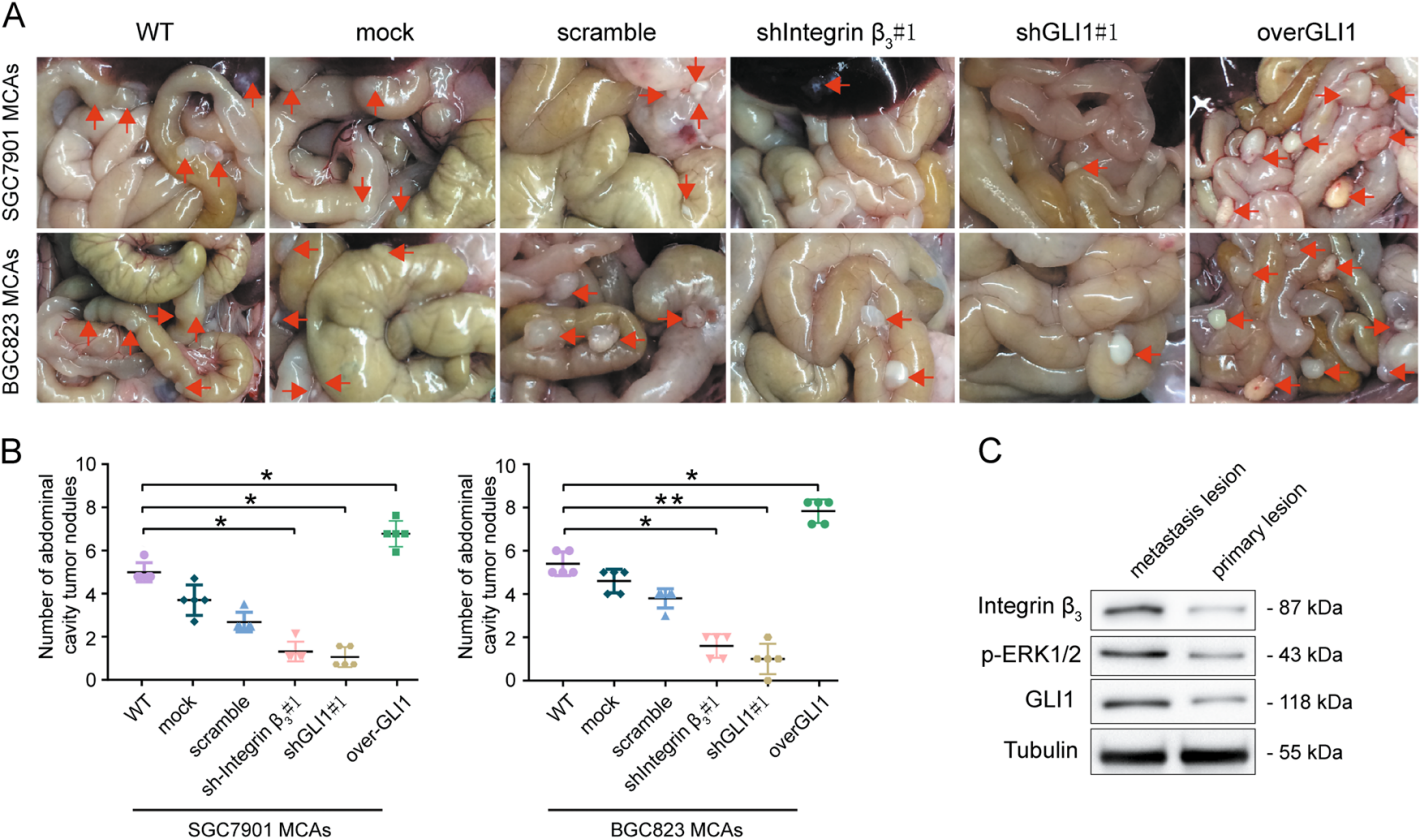
In vivo validation of the role of the Integrin α_v_β_3_/ERK1/2/GLI1 pathway on the peritoneal metastasis of GC. **a**, **b** Higher abdominal cavity tumor nodules formed by overGLI1 and lower nodules formed by shIntegrin β_3_#1 and shGLI1#1 compared to the control group transduced SGC7901 MCAs and BGC823 MCAs. Arrows indicate intraperitoreal nodules. **c** Western blottingting showing higher levels of Integrin α_v_β_3_, p-ERK1/2, and GLI1 in fresh surgical specimens of GC peritoneal metastatic compared to paired primary tumors. Each bar in the figure represents the mean ± SEM of triplicates. *p < 0.05; **p < 0.01

To further verify the role of the non-classical pathway Integrin α_v_β_3_/ERK1/2/GLI1 in the peritoneal metastasis of gastric cancer in vivo, we used scattered cells from BGC823 MCAs and SGC7901 MCAs transduced with either shIntegrin β_3_#1, shGLI1#1, negative control shCon, or transduced with overexpression vector of GLI1, empty vector, or a blank control to construct a model of peritoneal metastasis of gastric cancer in nude mice (Fig. 5a). The number of abdominal cavity tumor nodules were compared in each experimental group (Fig. 5b). The results showed that the number of xenograft tumors in the abdominal cavity of nude mice in the group of shIntegrin β_3_#1 and shGLI1#1 were significantly decreased, while the number in the overGLI1 group was significantly increased.

Subsequently, we performed Western blotting assays (Fig. 5c) on fresh surgical specimens of primary gastric tumors and peritoneal metastatic tumors from patients who had peritoneal metastasis after gastric cancer surgery. The results showed significant upregulation levels of Integrin α_v_β_3_, p-ERK1/2, and GLI1 in the specimens of GC peritoneal metastasis than primary lesions. This data further indirectly validates the potential role of the Integrin α_v_β_3_/ERK1/2/GLI1 pathway in the peritoneal metastasis of gastric cancer.

## Discussion

Here, we discovered a non-classical pathway Integrin α_v_β_3_/ERK1/2/GLI1. This pathway can regulate the stem cell-like phenotype of peritoneal MCAs of exfoliated GC cells. This finding may partially explain why MCAs of GC cells can proliferate in the peritoneal microenvironment.

MCAs are a special unit of cell aggregates discovered recently^9,24^. They are formed by the adhesion of approximately 10–100 tumor cells that are exfoliated from the extracellular matrix microenvironment of the primary tumor site, and enter the blood, lymph ducts or abdominal cavity^29,30^. As an independent structural and functional unit, the MCAs of GC cells play an important role in peritoneal metastasis^10^. However, the mechanism for how floating MCAs of exfoliated GC cells survive in the abdominal cavity remains unclear. We have shown that peritoneal MCAs of GC cells have strong ability of self-renewal, proliferation, drug resistance and tumorigenesis. Further experiments demonstrated corresponding changes in stemness-related markers, including Bmi1, Oct4, CD44 and ALDH1A1, which were significantly upregulated in floating MCAs of exfoliated GC cells. These results suggest that floating MCAs may have a stem cell-like phenotype. Interestingly, studies of human inflammatory breast cancer tissue samples showed that MCAs of vascular tumor thrombi may also have cancer stem cell-like characteristics^31^. Our previous study demonstrated that the expression of the stemness-related markers Bmi1, Oct4, CD44, and ALDH1A1 were significantly decreased after 1 week of adherent culture of scattered cells that were mechanically isolated from peritoneal MCAs of exfoliated GC cells (Supplementary Fig. S1f). This indicates that the tightly unique structure of MCAs may play an important role in the maintenance of stem cell-like phenotype, and the regulating mechanism needs to be explored.

Integrin is highly expressed on the surface of malignant tumor cells of various tissues and vascular endothelial cells. It is closely related to the early metastasis and poor prognosis of many types of tumors^32–38^. We found that Integrin α_v_β_3_ was highly expressed on peritoneal MCAs of exfoliated GC cells (Supplementary Fig. S1a-c). This result suggests that Integrin α_v_β_3_ mediates cell aggregates and maintains the viability of peritoneal MCAs in GC cells. Further study showed that the inhibition of Integrin α_v_β_3_ reduced the ability of proliferation, self-renewal, tumorigenesis in MCAs of GC cells, and the protein levels of Bmi1, Oct4, CD44 and ALDH1A1. This indicates that Integrin α_v_β_3_ not only mediates the intercellular adhesion in MCAs of GC cells but may also play an important role in the regulation of the stemness of MCAs. Other studies have also shown that Integrin α_7_-positive esophageal squamous cancer cells display a clear tumor stem-like characteristics and more prominent features of the epithelial-mesenchymal transition^39^. However, the mechanism that Integrin participates in stemness regulation remains unclear. Integrin plays a stemness-biological function by regulating downstream GLI1 in prostate cancer^16^, but the specific regulatory pathway and mechanism need to be further studied. We demonstrated GLI1 was positively correlated with Integrin α_v_β_3_ in MCAs of GC cells. Downregulation of Integrin β_3_ significantly decreased the protein levels of GLI1 and the stem cell-like phenotype of MCAs. GLI1 decreased when Integrin α_v_β_3_ was promoted but ERK was inhibited. The luciferase reporter gene assay showed that GLI1 expression was directly regulated by ERK1/2. These results suggest that the stemness of MCAs in GC cells was maintained by regulating the activity of GLI1 through the Integrin α_v_β_3_/ERK1/2 pathway.

Previous studies on GLI have mostly focused on the classical signaling pathway i.e., the Hh ligands-PTCH1-SMO axis. In addition to the classical signaling pathway, there is a crosstalk between the GLI family, especially GLI1, and other signaling pathways that regulate a variety of behaviors of solid tumors^14,40^. We discovered a non-classical pathway Integrin α_v_β_3_/ERK1/2/GLI1 in MCAs of GC cells. Previous studies have shown that there is crosstalk between the Hedgehog and the MAPK pathway^41–43^. This study showed that the expression of GLI1 was not significantly changed after inhibiting the Hedgehog pathway in the MCAs of GC cells, but the expression of GLI1 was significantly reduced after inhibiting the ERK pathway. These data suggest that although crosstalk with the classical Hedgehog signaling pathway exists, GLI1 is mainly regulated through the non-classical pathway Integrin α_v_β_3_/ERK1/2 in MCAs of GC cells. The phosphorylation of the serine/threonine residue in GLI1 enables GLI1 to exert its biological function through the regulation of the transcription of its downstream target gene Bmil et al.^44^. In this study, we transduced the MCAs of GC cells with knockdown and overexpressed lentivirus of GLI1 to investigate the stem cell-like phenotype. The results showed that GLI1, as a downstream key effector of crosstalk between the Integrin α_v_β_3_ pathway and the classical Hedgehog signaling pathway, plays an important role in the regulation of the stem cell-like biological characteristics of MCAs of GC cells.

**Fig. 6:**
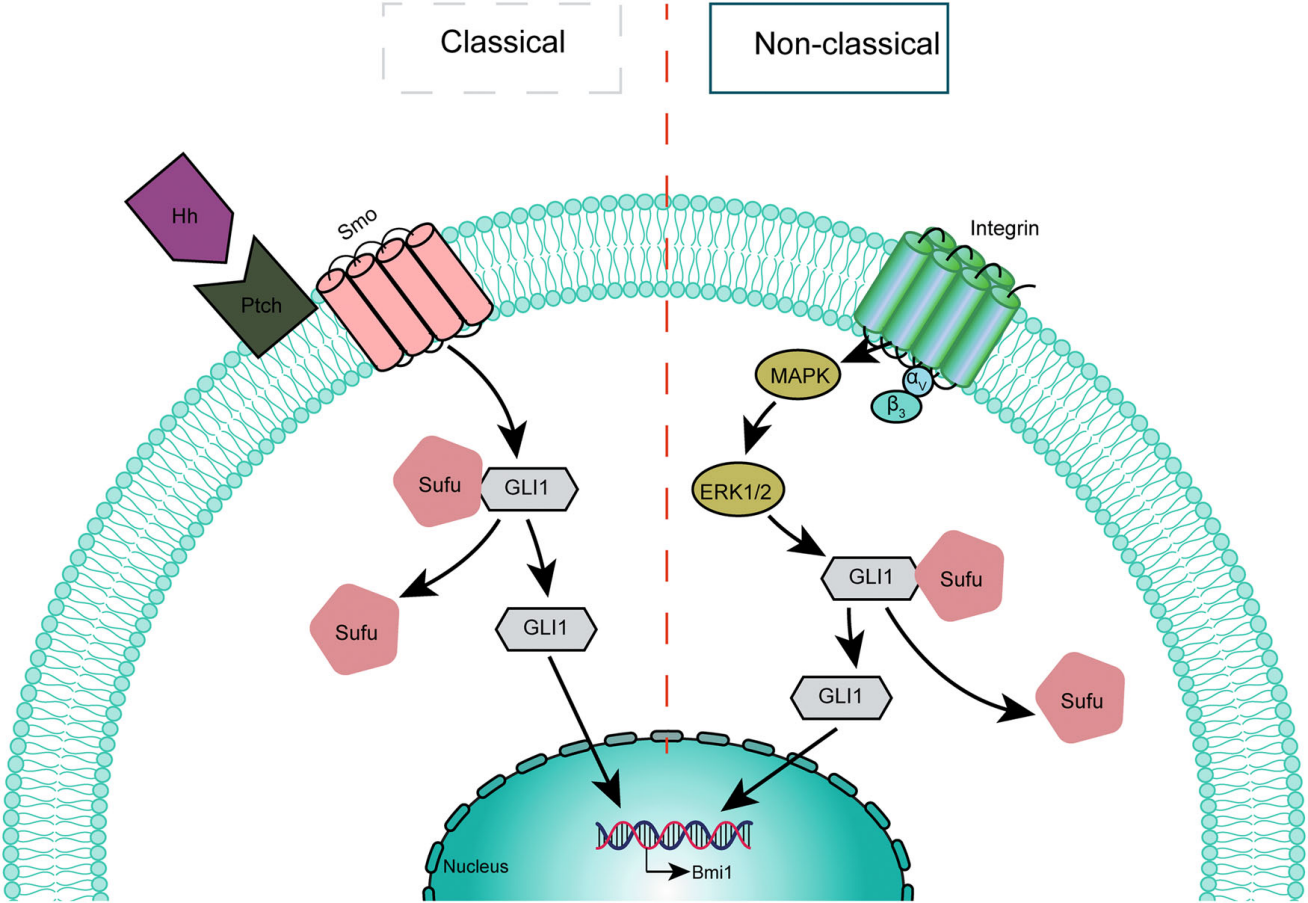
Integrin α_v_β_3_ mediates intercellular adhesion in MCAs of GC cells to form an independent functional unit and activates GLI1 through the non-classic ERK1/2 pathway and crosstalk with the classic Hedgehog pathway to maintain cancer stem cell-like phenotype

We demonstrated that Integrin α_v_β_3_ mediated intercellular adhesion in MCAs of GC cells to form independent functional units. Activated GLI1 through the non-classic Integrin α_v_β_3_/ERK1/2 pathway to initiate the transcription of downstream related target genes in order to maintain cancer stem cell-like phenotype of MCAs (Fig. 6). The maintenance of this stem cell-like phenotype may be based on the unique unitary structure of tight MCAs. If MCAs were separated into scattered cells, their stem cell-like phenotype may diminish or disappear. The new interpretation of the biological functions in MCAs of GC cells from the perspective of the characteristics of cancer stem cells can further reveal the important role of MCAs in tumor metastasis, which is of great significance in the prevention and treatment of GC peritoneal metastasis.

## Materials and methods

### Tissue specimens

Surgical specimens of primary GC tissues and corresponding peritoneal metastases tissues, ascites/peritoneal lavage fluid were obtained from 17 patients with GC between 2014 and 2019 (Southwest Hospital, Third Military Medical University, Chongqing, China). Abovementioned clinical information of patients was collected (Supplementary Table S1). No preoperative history of radiotherapy or chemotherapy was reported in any of the patients. Written consents for the biological studies were obtained from the patients or their guardians. According to the WHO standard^45^, each specimen was histologically examined and graded by two experienced pathologists. The acquisition and use of the specimens were approved by Institutional Research Ethics Committee.

### H&E

The ascites/peritoneal lavage fluid of GC patients was collected and centrifuged for 1500 RPM for 10 min, was fixed in 4% formaldehyde for 24 h, embedded in paraffin, and serially sectioned. The tissue sections were deparaffinized by washing twice in xylene (10 min each wash) and were rehydrated through a decreasing ethanol gradient (10 min per step). Subsequently, the tissue sections were washed with tap water for 10 min, incubated with 3% H_2_O_2_ at RT for 30 min, washed again with tap water for 5 min and immersed in PBS (0.01 mol/l, pH 7.4) for 5 min.

### Immunohistochemistry

IHC staining was performed according to the manufacturer’s instructions of the Dako REAL EnVision Detection System (Dako). The following are the primary antibodies used in IHC: CEA antibody (Cat#.ZM-0063, ASGB-BIO) or CK19 antibody (Cat#.ZM-0074, ASGB-BIO) or CD44 antibody (Cat#.ab51037, Abcam) or ALDH1A1 antibody (Cat#.ab52492, Abcam). Two pathologists in a blinded manner independently evaluated all slides.

### Cell culture and reagents

The isolation of peritoneal MCAs of exfoliated GC cells from ascites/peritoneal lavage fluid were referred to in the literature^25^. Human GC cell lines SGC7901 and BGC823 (originally purchased from ATCC) were authenticated using STR method and maintained in RPMI1640 medium (containing 10% FBS, Gibco) at 37 °C in 5% CO2 and 100% humidity. 2 × 10^4^ SGC7901 or BGC823 cells were seeded in 100-mm ultra-low adhesion dishes and cultured in the stem cell medium, i.e. serum free DMEM/F12 medium (1:1, Gibco, Grand island, USA) containing 20 μg/L epidermal growth factor (EGF, Sigma, USA), 20 μg/L basic fibroblast growth factor (Sigma) and B27 supplement (1X, Invitrogen, USA). The primary reagents used in this study were as follows: Integrin α_v_β_3_ inhibitor Cilengitide (Cat#.S7077, Selleck) or Integrin α_v_β_3_ co-stimulator ligand RGD (Arg-Gly-Asp) (Cat#.ab142698, Abcam) or ERK1/2 inhibitor PD-184161 (Cat#.ab143847, Abcam) or GLI1/2 inhibitor GANT61 (Cat.no.ab120904, Abcam) or Hedgehog/smoothen inhibitor Cyclopamine (Cat#.ab120392, Abcam).

### FACS analysis

GC cells were fixed with 4% paraformaldehyde for 15 min at room temperature andlabeled with a rabbit anti-human CD44 (1:30, Cat#.ab51037, Abcam) antibody or a rabbit anti-human ALDH1A1 (1:1000, Cat#.ab52492, Abcam) antibody for 30 min at 4 °C. An Alexa Fluor® 647 goat anti- rabbit IgG antibody was subsequently added for 30 min at 4 °C. The samples were analyzed by flow cytometry (BD FACSAria II, CA).

### Sphere formation assay

Threefold 1000 GC cells were seeded into ultra-low adhesion 96-well plates (Corning, USA) and propagated in the stem cell mediuma recommended by the manufacturer at 37 °C in 5% CO2 and 100% humidity. 20 μl stem cell medium was added to each well every 2 days. After incubation for 10 days, tumor spheres were counted to calculate the sphere formation efficiency.

### Cell-viability assay

The CCK8 assay was performed according to the manufacturer’s protocol (C0037, Beyotime, China). 2,000 cells per 100 ml medium were plated in 96-well plates. 12 hours later, the cells were treated with different CMs. At different time points, 10 ml of CCK8 solution was added to each well, and the wells were cultured at 37 °C in a humidified 5% CO2 atmosphere for 1 h. Then, the absorbance at 450 nm of each well was measured.

### Subcutaneous tumorigenicity and intraperitonealmetastasis assays

For subcutaneous tumorigenicity assay, the 6-week-old male nude mice were grouped randomly and double blindly (*n* = 6 in each group), then differently treated GC MCAs cells were injected subcutaneously into axilla of the mice. At the end of 6 weeks after the injection, the mice were killed. Xenograft tumors were removed and weighted. For intraperitoneal metastasis assay, 1 × 10^5^ GC MCAs cells were injected intraperitoneally into nude mice (*n* = 6 in each group). At the end of 4 weeks, the mice were killed and peritoneal metastatic foci were counted. GC MCAs cells dissociation and collection were conducted as previously described^46,47^. All animal procedures in this study were approved by Army Medical University (Third Military Medical University) Animal Committee.

### Colony formation assay

Colony formation assay was initiated by seeding 200 viable GC cells in each 6-well plates. Cells were cultured in the RPMI1640 medium (containing 10% FBS). After incubating for 2 weeks and staining with crystal violet, the colonies which contained more than 50 cells were counted.

### Western blot and immunoprecipitation

Immunoblotting analyses and Immunoprecipitation were performed as previously described^48,49^. The primary antibodies used in this study were as follows: anti-CD44 (Cat#.ab51037, Abcam) or anti-ALDH1A1 (Cat#.ab52492, Abcam) or anti-Bmi1 (Cat#.ab38295, Abcam) or anti-Oct4(Cat#.ab134218, Abcam) or anti-Integrin β_3_ (Cat#.ab17507, Abcam) or Phospho-ERK1/2 Antibody (Cat#.9101,CST) or anti-GlI1 (Cat#.ab92611, Abcam) or anti-Caspase-3 (Cat#. ab4051, Abcam) or anti-Ki67 (Cat#. No.AF0198, Affinity) or anti-Integrin β_5_ (Cat#. ab31327, Abcam) or anti-Smo (Cat#. No. DF5152, Affinity) or anti-Nanog (Cat#. ab14959, Abcam) or anti-c-Myc (Cat#.ab11917, Abcam) or anti-Cyclin D1 (Cat#. No. DF6386, Affinity) or anti-p-Thr/Ser (Cat#.ab17464, Abcam).

### Real-time PCR analysis

TRIzol Reagent (Thermo fisher) and PrimeScript RT Reagent kit (Takara Biomedicals, Kusatsu, Japan) were used to extract RNA and generate cDNA in accordance with the manufacturer’s instructions. Specific primers and SYBR Green I (Takara Biomedicals) were applied to perform Real-time PCR. Procedures of Ral-time PCR were conducted by BI 7500HT system. 2^−ΔΔCt^ method was applied to quantify target gene relative expression. The specific primers were shown in Primers and Oligonucleotides in Supplementary Table S4.

### Knockdown and overexpression in GC cells

The sequences containing an effective shRNA-targeting Integrin β_3_ or GLI1 or Integrin β_5_ or Smo and corresponding scrambles were listed in Supplementary Table S2-5. Lentivirus particles containing shIntegrin β_3_ or shGLI1 and scrambles were prepared by Hanbio Biotechnology or Shanghai GeneChem Co., Ltd. and used to infect SGC7901 and BGC823 GC cells. Then, stably transduced GC cells were selected using FACS. For overexpressing GLI1 in GC cells, lentiviral particles containing human full-length GLI1 was prepared and used to infect SGC7901 and BGC823 cells. Stable GLI1 overexpressing (over GLI1) and control cells were selected using 3 μg/ml puromycin.

### Statistical analysis

The results from representative experiments are presented. Student’s t-test and one-way ANOVA were used to compare the means of two or more groups. SPSS 20.0 software (SPSS Inc., Chicago, IL, USA) and GraphPad Prism 7 (GraphPad, La Jolla, CA, USA) were used for statistical analysis. All experiments were conducted at least three times. *p* < 0.05 was considered as statistically significant.

## Supplementary information

Supplementary Material
