# Supplementary material for: GLI1 activation by non-classical pathway integrin αvβ3/ERK1/2 maintains stem cell-like phenotype of multicellular aggregates in gastric cancer peritoneal metastasis
Source: Cell Death Dis. 2019 Jul 31;10(8):574. doi: 10.1038/s41419-019-1776-x (PMC6668446; doi:10.1038/s41419-019-1776-x)
Supplement: Supplementary file 1 — Supplementary Material [file 41419_2019_1776_MOESM1_ESM.docx]

**Supplementary Tables**

**Table S1**. Clinicopathological characteristics of patients with GC

| Clinical Characteristic | Number | Percentage (%) |
| --- | --- | --- |
| Age |  |  |
| ≥60 | 8 | 47 |
| <60 | 9 | 53 |
| Gender |  |  |
| Male | 10 | 59 |
| Female | 7 | 41 |
| Histology grade |  |  |
| G1  G2 | 2  6 | 12  35 |
| G3 | 9 | 53 |
| Extent of the Tumor |  |  |
| Tis-T1  T2 | 0  0 | 0  0 |
| T3  T4 | 7  10 | 41  59 |
| Lymph Nodes Status |  |  |
| N1  N2  N3 | 3  6  8 | 18  35  47 |

**Table S2.** Sequences of GLI1 shRNAs and control shRNAs

| Name | Target Sequence | GC% |
| --- | --- | --- |
| sh GLI1#1 | GAAGTCTGAGCTGGACATGCT | 52.4% |
| sh GLI1#2 | GTTCACATGCGCAGACACACT | 52.4% |
| sh GLI1#3 | CAGCATCACTGAGAATGCTGCCAT | 50% |
| sh Control | TTCTCCGAACGTGTCACGTAA | 47.6% |

**Table S3.** Sequences of Integrin β_3_ shRNAs and control shRNAs

| Name | Target Sequence | GC% |
| --- | --- | --- |
| sh Integrin β_3_#1 | CGTCAGATTCCAGTACTAT | 42.1% |
| sh Integrin β_3_#2 | ACGTCTACCTTCACCAATA | 42.1% |
| sh Integrin β_3_#3 | CATATAGCATTGGACGGAA | 42.1% |
| sh Control | TTCTCCGAACGTGTCACGT | 52.6% |

**Table S4.** Sequences of Smo shRNAs and control shRNAs

| Name | Target Sequence | GC% |
| --- | --- | --- |
| sh Smo #1 | CCTGCTGTTATTCTCTTCTACGTCA | 44% |
| sh Smo #2 | TCATTACCTTCAGCTGCCACTTCTA | 44% |
| sh Smo #3 | TGACCTCAATGAGCCCTCAGCTGAT | 50% |
| sh Control | TTCTCCGAACGTGTCACGTAA | 47.6% |

**Table S5.** Sequences of Integrin β_5_ shRNAs and control shRNAs

| Name | Target Sequence | GC% |
| --- | --- | --- |
| sh Integrin β_5_ #1 | CCTTTCTGTGAGTGCGACAACTTCT | 48% |
| sh Integrin β_5_ #2 | CAGGAGGCTGTGCTATGTTTCTACA | 48% |
| sh Integrin β_5_ #3 | CCGCTATGAAATGGCTTCAAATCCA | 44% |
| sh Control | TTCTCCGAACGTGTCACGTAA | 47.6% |

**Table S6.** The primer sequences for qRT-PCR in the experiments

| Bmi1- Forward | CTCCACCTCTTCTTGTTTG |
| --- | --- |
| Bmi1- Reverse | CTGATGACCCATTTACTGAT |
| Oct4- Forward | AGGGCAAGCGATCAAGCA |
| Oct4- Reverse | CAGGGAAAGGGACTGAGGAG |
| CD44- Forward | GATGGCACCCGCTATGT |
| CD44- Reverse | ATCCAGGGACTGTCTTCGT |
| ALDH1A1- Forward | ATGTCATCCTCAGGCACG |
| ALDH1A1- Reverse | ATCCTCCTTATCTCCTTCTTCTA |
| Nanog- Forward | GATCGGGCCCGCCACCATGAGTGTGGATCCAGCTTG |
| Nanog- Reverse | GATCGAGCTCCATCTTCACACGTCTTCAGGTTG |
| GLI1- Forward | ACCTTCCTACCAGAGTCCCAAGT |
| GLI1- Reverse | CCCTATGTGAAGCCCTATTTGC |
| Actin- Forward | CATTCCAAATATGAGATGCGTTGT |
| Actin- Reverse | TGTGGACTTGGGAGAGGACT |

**Supplementary Figure S1**

**
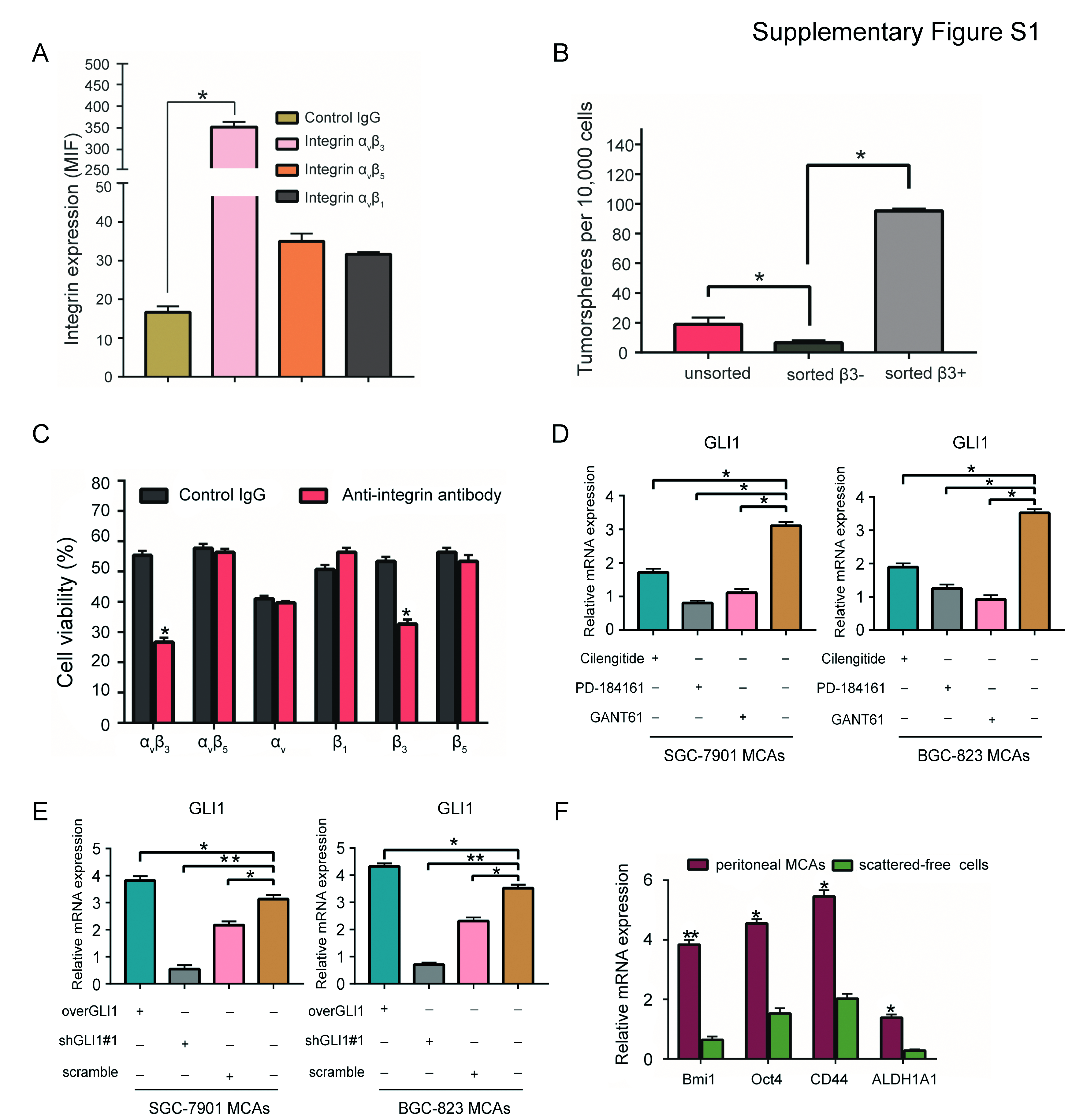
**

**Fig. S1 a** Flow cytometry-based cell sorting showing higher Integrin α_v_β_3_ level compared with other subunits in peritoneal MCAs of exfoliated GC cells. **b** Increased tumor spheres formating ability of Integrin β_3_+ group compared with Integrin β_3_^--^ group in peritoneal MACs of exfoliated GC cells. **c** Decreased cell viability of peritoneal MACs of exfoliated GC cells treated with the blocking antibody of Integrin α_v_β_3_. **d** Real-time PCR showing decreased GLI1 in each group of SGC7901 MCAs and BGC823 MCAs after inhibiting Integrin α_v_β_3_, ERK, and GLI1 compared with the blank control group. **e** Real-time PCR showing upregulated GLI1 in the overGLI1 group and downregulated GLI1 in the shGLI1#1 group compared with the control group in SGC7901 MCAs and BGC823 MCAs**. f** Real-time PCR showing upregulated Bmi1, Oct4, CD44, and ALDH1A1 in peritoneal MCAs of exfoliated GC cells compared with scattered-free cells. Each bar in the figure represents the mean ± SEM of triplicates. *p < 0.05，**p < 0.01.

**Supplementary Figure S2**


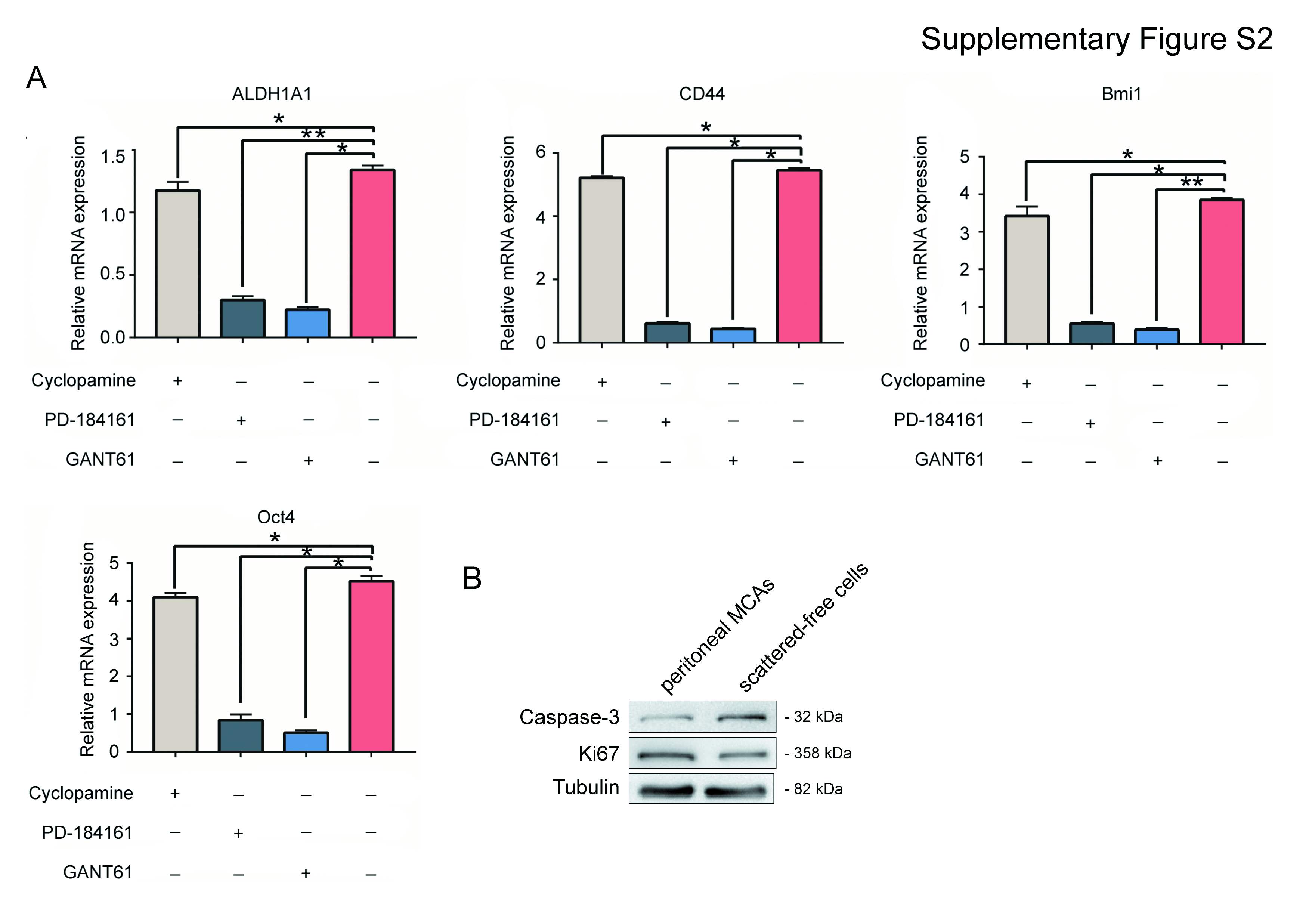


**Fig. S2 a** Real-time PCR showing significantly decreased stemness-related genes ALDH1A1, CD44, Bmi1, Oct4 in peritoneal MCAs of exfoliated GC cells treated with ERK1/2 inhibitor PD-184161 or GlI1 inhibitor GANT61 compared with Hedgehog pathway inhibitor Cyclopamine. **b** Western blottingting showing upregulated Ki67 and downregulated Caspase-3 expressed by peritoneal MCAs compared to scattered-free cancer cells in the control group. Each bar in the figure represents the mean ± SEM of triplicates. *p < 0.05，**p < 0.01.

**Supplementary Figure S3**


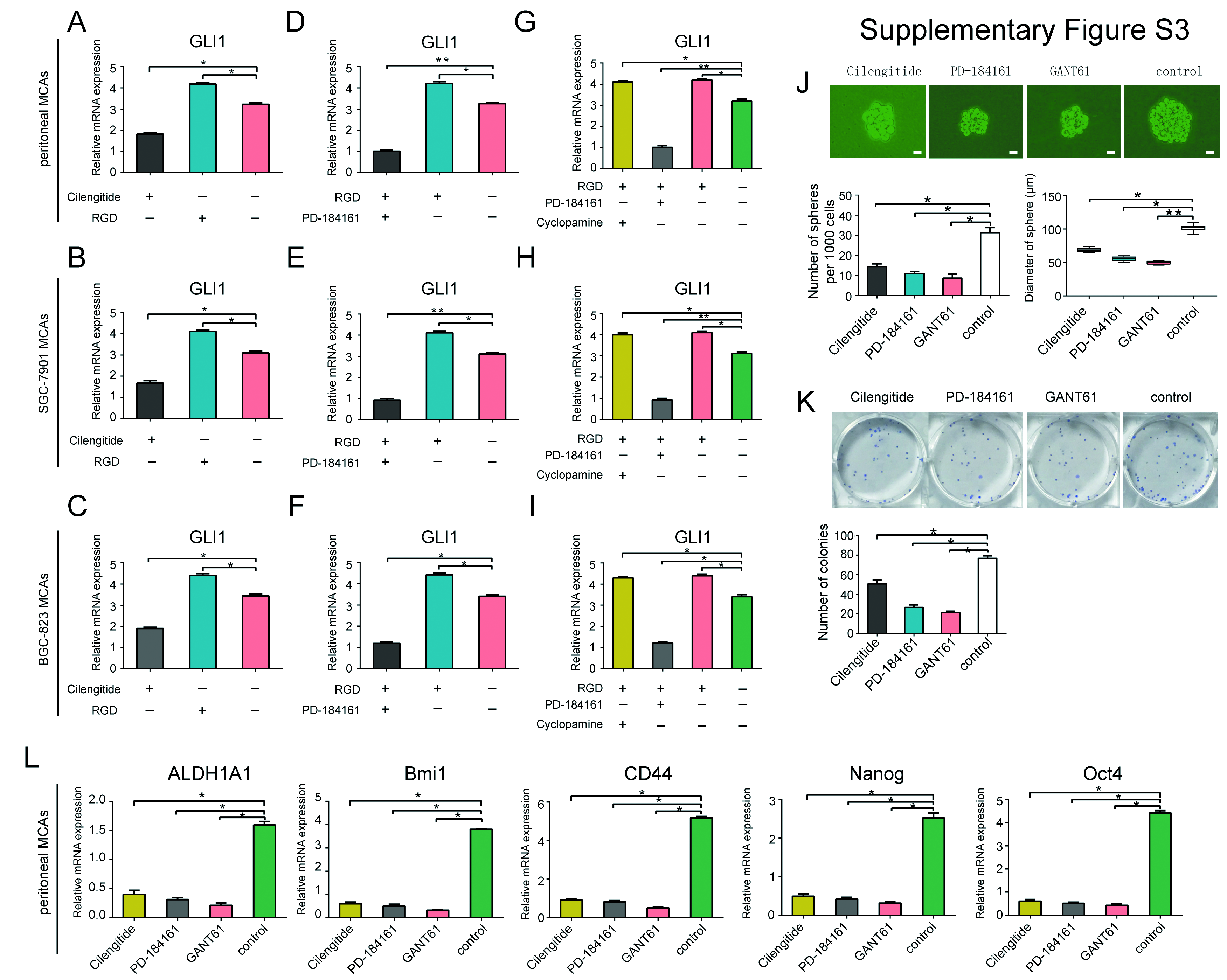


**Fig. S3 a-c** Real-time PCR showing downregulated GLI1 with Integrin αvβ3 inhibitor Cilengitide and upregulated GLI1 with co-stimulator ligand RGD compared to the blank control in peritoneal MCAs, SGC7901 MCAs and BGC823 MCAs. **b-f** Real-time PCR showing downregulated GLI1 with Integrin αvβ3 co-stimulator ligand RGD plus ERK1/2 inhibitor PD-184161 and upregulated GLI1 with RGD alone compared to the blank control in peritoneal MCAs , SGC7901 MCAs and BGC823 MCAs. **g-I** Real-time PCR showing decreased showing slightly decreased GLI1 with Integrin αvβ3 co-stimulator ligand RGD plus Hedgehog/Smoothened pathway inhibitor Cyclopamine compared with the group of RGD alone in peritoneal MCAs, SGC7901 MCAs and BGC823 MCAs. **j** Decreased tumor spheres forming ability in the group of Integrin α_v_β_3_ inhibitor Cilengitide or ERK1/2 inhibitor PD-184161 or GLI1 inhibitor GANT61 compared to the blank control in peritoneal MCAs. Scale bar = 10μm. **k** Lower colony forming ability in the group of Integrin α_v_β_3_ inhibitor Cilengitide or ERK1/2 inhibitor PD-184161 or GLI1 inhibitor GANT61 compared to the blank control in SGC7901 MCAs. **l** Real-time PCR showing downregulated stemness-related genes CD44, ALDH1A1, Oct4, Nanog and Bmi1 in the group of Integrin α_v_β_3_ inhibitor Cilengitide or ERK1/2 inhibitor PD-184161 or GLI1 inhibitor GANT61 compared to the blank control in SGC7901 MCAs. Each bar in the figure represents the mean ± SEM of triplicates. *p < 0.05,**p < 0.01.

**Supplementary Figure S4**


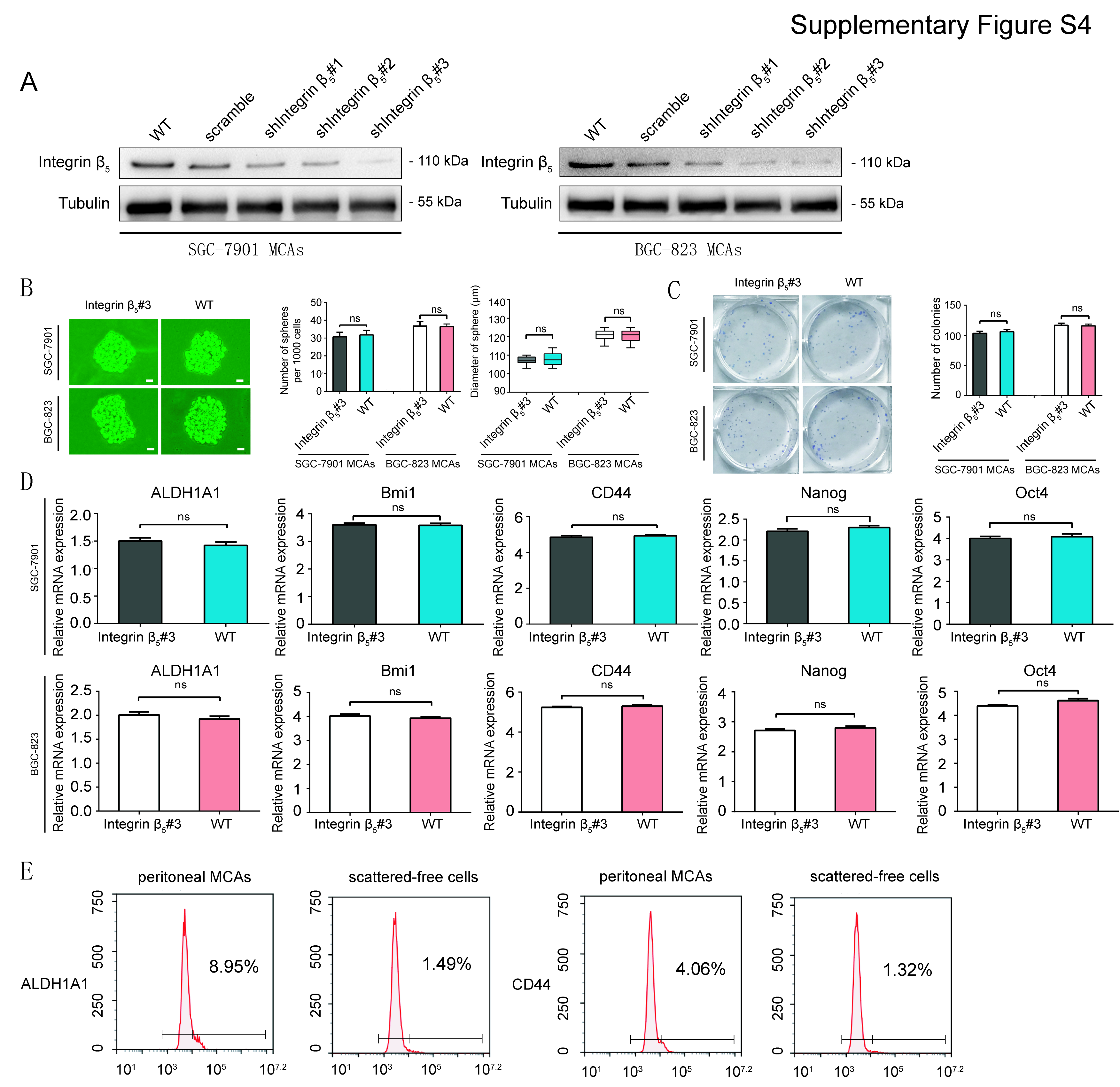


**Fig. S4 a** Western blottingting showing the most pronounced effect of Integrin β_5_ silencing in the shIntegrin β_5_#3 group in BGC823MCAs and SGC7901MCAs. **b** Similar tumor spheres forming ability in the shIntegrin β_5_#3 group and the control group (WT) in SGC7901 MCAs and BGC823 MCAs. Scale bar = 10μm. **c** Similar colony forming ability in the shIntegrin β_5_#3 group and the control group (WT) in SGC7901 MCAs and BGC823 MCAs. **d** Real-time PCR showing similar stemness-related genes CD44, ALDH1A1, Oct4, Nanog and Bmi1 in the shIntegrin β_5_#3 group and the control group (WT) in SGC7901 MCAs and BGC823 MCAs. **e** Flow cytometry-based cell sorting showing higher ALDH1A1 and CD44 level compared with scattered-free cells in peritoneal MCAs of exfoliated GC cells. Each bar in the figure represents the mean ± SEM of triplicates. ns, no significance; *p < 0.05,**p < 0.01.

**Supplementary Figure S5**


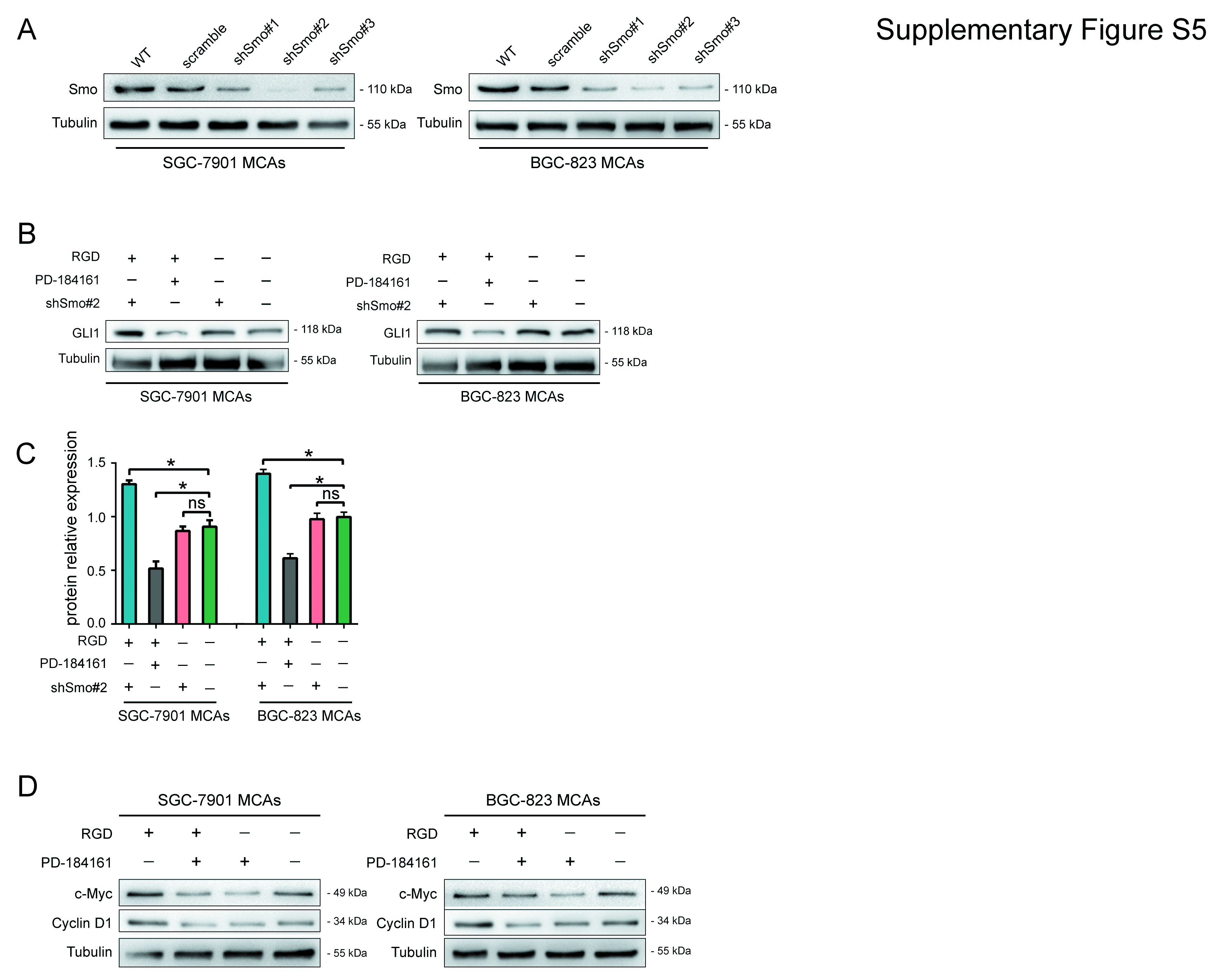


**Fig. S5 a** Western blottingting showing the most pronounced effect of Smo silencing in the shSmo #2 group in BGC823MCAs and SGC7901MCAs. **b,c** Western blottingting and quantitative analysis showing the protein levels of GLI1 in the shSmo #2 groups did not decrease compared with the blank control group in SGC7901 MCAs and BGC823 MCAs. **d** Western blottingting showing decreased c-Myc and Cyclin D1 in the group of Integrin α_v_β_3_ co-simulator ligand RGD plus ERK1/2 inhibitor PD-184161 or PD-184161 alone compared to the blank control. Each bar in the figure represents the mean ± SEM of triplicates. ns, no significance; *p < 0.05,**p < 0.01.

**Supplementary Figure S6**


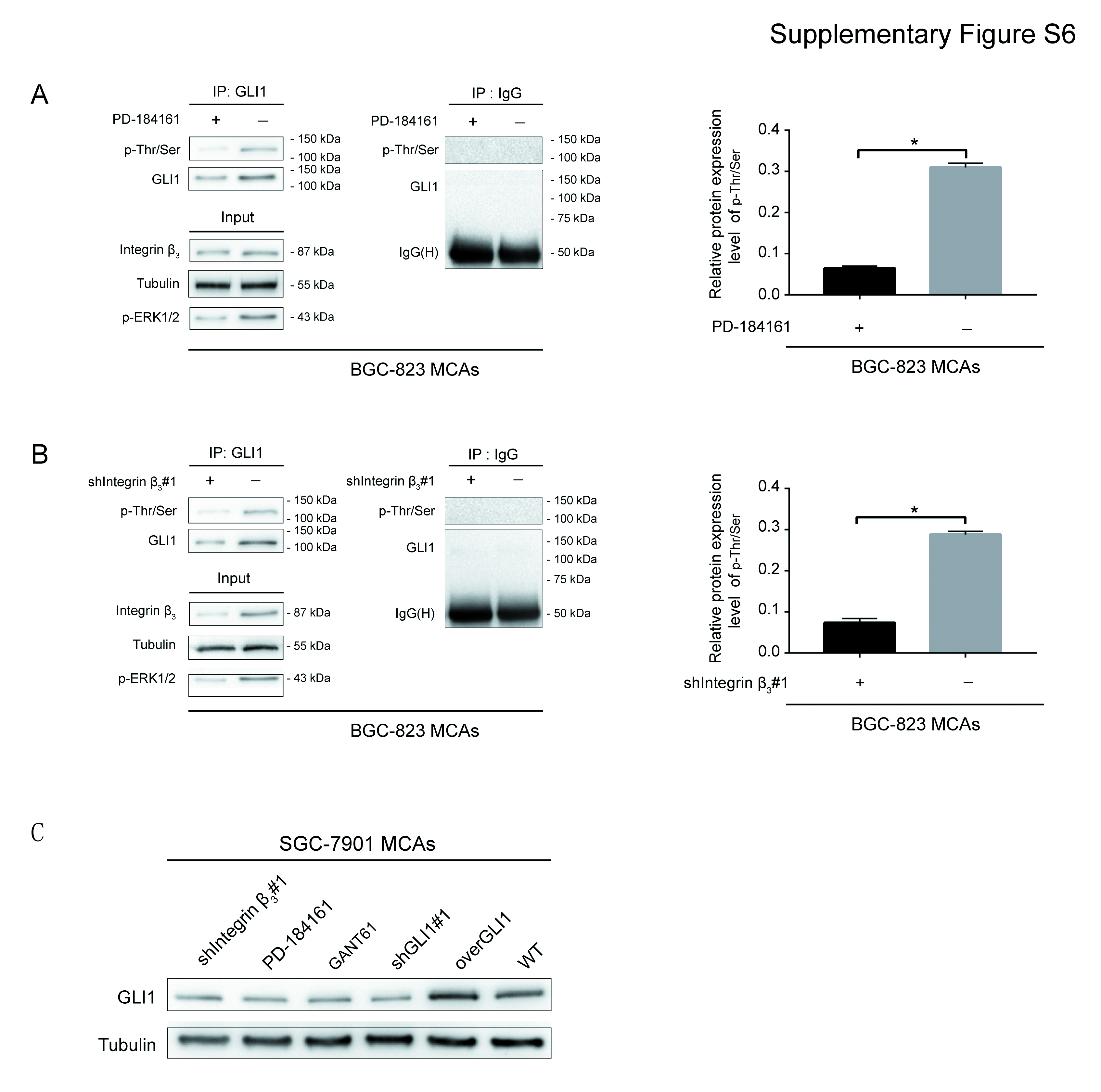


**Fig. S6 a** Immunoprecipitation showing decreased GLI1 phosphorylation in BGC-823 MCA cells treated with the selective ERK1/2 inhibitor compared to the blank control. Band intensities for p-Thr/Ser expression were quantified and normalized to tubulin. **b** Immunoprecipitation showing decreased GLI1 phosphorylation in the shIntegrin β_3_#1 group compared to the blank control in BGC-823 MCA cells. Band intensities for p-Thr/Ser expression were quantified and normalized to tubulin. **c** Western blottingting showing downregulated GLI1 in the group of shIntegrin β_3_#1 or ERK1/2 inhibitor PD-184161 or GLI1 inhibitor GANT61 or shGLI1#1, and upregulated GLI1 in the group of overGLI1 compared to the blank control in SGC7901 MCAs. Each bar in the figure represents the mean ± SEM of triplicates. *p < 0.05,**p < 0.01.

Detailed Attribution of Authorship

1. **The contributions of each author**

Bo Tang and Peiwu Yu conceived and designed experiments;

Hui Dong carried out biochemical and molecular biology experiments;

Hongchang Liu carried out animal experiments;

Hui Dong and Wen Zhou helped write the manuscript;

Fan Zhang, Chuan Li, Jun Chen, Chenjun Tan participated in statistical analysis.

**2. The author contributions to each figure**

In Figure 1, Hui Dong generated the western blot data, the H&E and immunohistochemistry images; Bo Tang generated the data of Cell colony formation and CCK8 assays; Hongchang Liu generated animal experiments data; Fan Zhang analyzed the data and Wen Zhou assembled the figure;

In Figure 2, Bo Tang generated the western blot data; Chuan Li analyzed the data and Wen Zhou assembled the figure;

In Figure 3, Hui Dong generated the data of proliferation, self-renewal, the protein levels of stemness-related markers; Hongchang Liu generated animal experiments data; Jun Chen analyzed the data and Wen Zhou assembled the figure;

In Figure 4, Peiwu Yu generated the western blot data; Hui Dong generated the data of proliferation, self-renewal; Hongchang Liu generated animal experiments data; Chenjun Tan analyzed the data and Peiwu Yu assembled the figure;

In Figure 5, Hui Dong generated the western blot data; Hongchang Liu generated animal experiments data; Jun Chen analyzed the data and Bo Tang assembled the figure;

In Figure 6, Bo Tang assembled the figure;

In Figure S1-S6, Hui Dong generated the data of Real-time PCR and western blot; Chuan Li analyzed the data of Flow cytometry-based cell sorting and Peiwu Yu assembled the figure;
